# How different cystoscopy methods influence patient sexual satisfaction, anxiety, and depression levels: a randomized prospective trial

**DOI:** 10.1007/s11136-016-1493-1

**Published:** 2017-01-03

**Authors:** Wojciech Krajewski, Katarzyna Kościelska-Kasprzak, Joanna Rymaszewska, Romuald Zdrojowy

**Affiliations:** 1 Department of Urology and Oncological Urology, Wrocław Medical University, Borowska 213, 50-556 Wrocław, Poland; 2 Department of Nephrology and Transplantation Medicine, Wroclaw Medical University, Borowska 213, 50-556 Wroclaw, Poland; 3 Department of Psychiatry, Wrocław Medical University, ul. Pasteura 10, 50-367 Wrocław, Poland

**Keywords:** Cystoscopy, Bladder cancer, Sexual satisfaction, Depression, Anxiety, Pain

## Abstract

**Purpose:**

Bladder cancer (BC) is one of the most common cancers worldwide. BC diagnosis and surveillance is based on cystoscopy (CS). CS impact on patient’s depression, anxiety, and sexual satisfaction (SS) is not sufficiently studied. There are no data on patient’s comfort with flexible or rigid CS.

**Methods:**

We prospectively evaluated pain perception (PP), depression, anxiety, and SS of 100 male patients who previously underwent at least one rigid CS in our department as surveillance after TURB procedure due to non-muscle-invasive BC and were scheduled for the next CS examination. The patients were randomized for flexible or rigid CS. Before CS, patients described their recalled rigid CS-related pain by NRS and fulfilled HADS and SS questionnaires. After CS, PP was re-evaluated immediately and HADS and SS within 7–10 days following the CS.

**Results:**

The baseline scores include 5.2 ± 2.6 points for rigid CS recalled pain, 7.2 ± 3.0 points for HADS anxiety, 5.8 ± 3.5 for depression, and 27.8 ± 5.1 for SS. The flexible CS-related pain was approximately three times lower than the recalled pain level and also than the current rigid CS related (*p* < 0.001). Mean SS score was two points lowered after rigid CS (*p* < 0.001). One point decrease in anxiety level was observed after flexible CS (*p* < 0.001). Multivariate analysis supported the hypothesis of patients benefiting from flexible CS in terms of pain perception, anxiety symptoms, and SS.

**Conclusions:**

Our study demonstrates the superiority of flexible CS in terms of pain alleviation, and shifts in SS and anxiety levels.

## Introduction

Bladder cancer (BC) is one of the most common and lethal cancers worldwide [1, 2]. BC diagnosis and surveillance is mainly based on endoscopic cystoscopy (CS). Despite BC diagnosis, CS is commonly used by the urologists to evaluate haematuria, voiding symptoms, and for minor procedures, such as foreign body removal.

CS is a procedure, which allows visualization of the urethra and the bladder walls. There are two main types of CS: rigid and flexible. Traditionally, CS is performed by means of a rigid, hollow, steel tube (cystoscope) equipped with a lens, that is introduced through the urethra into the bladder. Flexible CS, similarly to gastroscopy or colonoscopy, is based on flexible composite cystoscopes which have actively deflecting tip. Elastic body of flexible cystoscope adapts to a certain extent to the contour of the lower urinary tract and, therefore, is less traumatic. Both flexible and rigid tools were demonstrated to have equal efficacy [3, 4]. Rigid tools offer good irrigant flow and handiness along with wide working channel lumen. Flexible cystoscopes provide more possibilities for patient positioning, enable easy course over an elevated bladder neck or median lobe, facilitate full inspection of the bladder mucosa, and, what is the most important, significantly improve patient comfort [5]. Urologists often repeat an argument that rigid cystoscopes offer a better image quality. However, with the video HD cystoscopes that have been introduced recently, the image quality greatly exceeds the one obtained by the traditional rigid tools.

Despite the evident CS benefits, it has been shown that CS is an invasive procedure that is perceived as burdensome and is associated with pain and discomfort [6–8]. Because of that, in some cases, discomfort leads to the avoidance or total abandonment of the cystoscopic surveillance.

It is crucial to concern quality of life in patients with BC, as BC is a disease which always requires long, staged, repeated and mutilating treatment with a life time period of cancer monitoring [9]. The consequences of manipulation on genitourinary tract include psychological problems, such as anxiety about losing masculinity, lowered self-esteem, and lowered sense of sexuality, anxiety about the quality of intimate relationships with a partner, and lack of acceptance of one’s body.

Only a few studies analysing patient ailments during and after CS are available. However, they are based on heterogeneous groups of patients and/or disorders and they are mainly retrospective or cross-sectional studies [10–12]. Most literature concerning patient quality of life or mental health addresses muscle-invasive BC patients, who previously underwent radical cystectomy. These studies focus on the effect of different urinary diversion methods [13–15]. Only a few analyse patients with non-muscle-invasive bladder cancer (NMIBC) [16, 17].

There are not enough objective data regarding CS impact on patient’s depression, anxiety, and sexual satisfaction. There are also no data to demonstrate authoritatively superiority of either flexible or rigid CS. We neither know how the psychological features affect pain perception during CS [12, 18].

### Aim

In this study, we prospectively evaluated pain perception and shift in depression, anxiety, and sexual satisfaction (SS) levels in men undergoing cyclic CS assessment with rigid and flexible instruments after transurethral resection of non-muscle-invasive bladder tumour (TURB).

## Methods

An institutional ethics committee approved the study, and all patients gave written informed consent.

### Study design

We prospectively evaluated pain perception, depression, anxiety, and sexual satisfaction of male patients who underwent at least one rigid CS at the Urology and Urologic Oncology Department of Wroclaw Medical University as regular surveillance after TURB procedure due to NMIBC and who were scheduled for the next CS procedure. Exclusion criteria involved patients under 18 years, with indwelling catheters, with history of any but TURB surgery on genitourinary tract, CS with intervention, symptomatic urinary tract infection, and inability to cooperate with psychological evaluations. Patients taking medications that could affect their mental states were also disqualified.

All male patients that were scheduled for cystoscopy in our department and who meet inclusion criteria were invited to the study. Twenty three patients were not able to fill the initial questionnaires, and they were not included in the randomization process. Those, who did not provide SS responses, but filled HADS and PP questionnaires, were included in the study. The patients were randomly appointed to undergo flexible CS (group F) or rigid CS (group R). We used a statistical package to generate a random group assignment for the prospective enrolment. The study was single-blinded; patients did not know which procedure will be performed at the time they completed the first set of questionnaires. Seventeen patients did not resend the second set of the questionnaires and were excluded from the study. The study enrolment was stopped after both the study groups reached the expected size of 50 participants (100 patients in total).

### Cystoscopy

All procedures were performed at the Urology and Urologic Oncology Department of Wrocław Medical University. One urologist (WK) conducted all flexible procedures and the majority of rigid CSs. All CSs were performed in the dorsal lithotomy position. All CSs started with routine disinfection of external genitalia and perineum with antiseptics agent. Instillation of a lubricating gel containing 2% lidocaine was performed at least 5 min before endoscope insertion. All CSs were carried out without any systemic sedation or analgesia. All endoscopes were introduced and advanced under direct operator vision. Rigid Storz 20 Fr and flexible 15 Fr ACMI cystourethroscope were used. Patients had the opportunity to observe CS on the screen. No antimicrobial prophylaxis was used routinely.

### Statistical analysis

Based on the preliminary assessment of the NMIBC patients (data not shown), 100 patients were calculated to be sufficient to observe at least 25% decrease in pain level after flexible CS, assuming alpha 0.05 and power of 0.80.

The numerical results are presented as mean ± standard deviation. Group comparison was analysed with Mann–Whitney test. The paired observations, pre- and post-CSs, were compared using Wilcoxon signed-rank test. The variations of frequencies were tested using *χ* ^2^. The correlations were assessed with Spearman or gamma correlation. The *r*_*s*_ values of 0.20–0.39 were considered weak, 0.40–0.59 moderate, and >0.60 strong associations. *γ* of 0.25–0.49 was considered a weak, 0.50–0.74 a moderate, and >0.75 a strong relationship.

The Bonferroni correction for multiple testing was applied when appropriate, and the adjusted *p* values are shown in the paper. The value of adjusted *p* < 0.05 was considered statistically significant.

General regression model of ANCOVA was used for multivariate analysis. The type of cystoscope used during the study procedure was modelled as 1 for rigid and 0 for flexible one.

All calculations were performed with Statistica v.12 (Statsoft, Poland).

### Pain perception

Patient’s pain perception was assessed with numeric rating scale (NRS) [19]. An 11-point scale, ranging from 0 (free from pain) to 10 points (unbearable pain), was used. Before the CS procedure, patients were asked to evaluate the recalled pain related to the previous rigid CS. Pain experienced during the current CS was estimated within a few minutes after the CS.

### Psychological questionnaires

The questionnaires were completed before CS in a room that provided privacy. After CS, patients were discharged with an addressed and stamped envelope containing the second copy of questionnaires, which was to be completed 7–10 days following the CS and resent to our department. Patients who did not complete Hospital Anxiety and Depression Scale (HADS) questionnaires or completed HADS/SS questionnaires partially were excluded from the analyses. Patients who did not resend copies of surveys were also disqualified.

#### Hospital anxiety and depression scale (HADS)

Anxiety and depression were measured with the validated ‘Hospital Anxiety and Depression Scale’ (HADS) [20]. The HADS, presented by Zigmond and Snaith, has been translated, tested, and used in various languages, including Polish [21]. This is a widely used tool, suitable for different kinds of patients, including those with both cancerous and non-cancerous diseases. As the items of the questionnaire are not concentrated on severe symptomatology, the scale is applicable to the general population samples [22].

The HADS consists of two subscales with seven items scored 0–3 each, rating anxiety (HADS-A) and depression (HADS-D), respectively. Based on individual sums of scores, patients can be assigned to one of the three categories: non-case/normal (0–7), borderline case (8–10), and definite case (11–21) for both depression and anxiety. Due to high sensitivity and specificity in detecting the generalized anxiety and depression symptoms, HADS is one of the most frequently used screening tools in medical conditions [21, 23]. HADS is a valid and reliable screening instrument both on aggregate European level and on country-specific level and can, therefore, be administered to patients across languages, countries, and institutions [24, 25]. The polish version of HADS conducted on gynaecological patients presented good internal consistency with Cronbach’s coefficient alpha at 0.84 and 0.78 for depression and anxiety subscales, respectively, and 0.88 for the whole questionnaire [21].

#### The sexual satisfaction questionnaire

Sexual satisfaction was measured with The Sexual Satisfaction Questionnaire designed by Nomejko and Dolińska-Zygmunt. This tool was designed for adults at all ages and it allows to measure a patient’s attitude (cognitive and emotional) towards their own sexual activity [26]. The Sexual Satisfaction Questionnaire is used to measure the sense of sexual satisfaction associated with two areas: sexual attractiveness of the individual and sexual activity. The questionnaire allows to look at human sexuality from the perspective of life’s quality of [27]. The questionnaire consists of ten statements, and the answers are provided using four-point Likert scale: 1—absolutely not, 2—rather not, 3—rather yes, and 4—definitely yes [28] (Table 1). A result of the questionnaire is the sum of points. Questions 1, 3, 5, 8, and 9 are encoded inversely. The theoretical distribution of the results fits within the 10–40 range. The method does not have the reference levels. The result is interpreted in relation to the examined population.

**Table 1:** Sexual Satisfaction Questionnaire questions [28]

1. I am disconcerted with a part of my sexual life
2. Sex is a source of pleasure for me
3. Thinking about sex generates negative emotions
4. I feel sexually attractive
5. I find myself a poor sexual partner
6. I do not have any problems in my sexual life
7. I like thinking about my sexual life
8. My sexual life frustrates me
9. I am afraid I do not satisfy my sexual partner
10. I find my sexual life fulfilling

It was shown by that SS level measured by SS questionnaire is positively correlated with the quality of life. The *r*-Pearson coefficient of SS and global quality of life correlation was: *r*  = 0.453; *p* < 0.001. In addition, the SS questionnaire presented good internal consistency with Cronbach’s coefficient alpha at 0.83 [26].

## Results

The study involved 100 male NMIBC patients who were scheduled for the consecutive cystoscopy as a regular surveillance after TURB procedure. The patients who fulfilled the study criteria were randomly assigned into rigid or flexible CS group differing in CS procedure applied. The groups were proved to be age and baseline scores matched (Table 2).

**Table 2:** Baseline study group characteristics

	Combined	Rigid CS	Flexible CS	Group comparison, *p* value
Age	69.0 ± 7.3 years [48 ÷ 86 years]	68.5 ± 7.8 years [48 ÷ 86 years]	69.5 ± 6.8 years [55 ÷ 83 years]	*p* = 1.000^a^
Pain perception	5.7 ± 2.4 [0 ÷ 10]	5.5 ± 2.8 [0 ÷ 10]	5.9 ± 2.0 [2 ÷ 10]	*p* = 1.000^a^
HADS—anxiety	7.2 ± 3.0 [1 ÷ 13]	7.5 ± 3.0 [1 ÷ 13]	6.8 ± 3.0 [1 ÷ 13]	*p* = 1.000^a^
HADS—anxiety qualitative				*p* = 0.944^b^
Borderline	32% [32/100]	36% [18/50]	28% [14/50]	
Definite	18% [15/100]	18% [9/50]	12% [6/50]	
HADS—depression	5.8 ± 3.5 [0 ÷ 14]	5.9 ± 3.7 [0 ÷ 14]	5.7 ± 3.3 [0 ÷ 12]	*p* = 1.000^a^
HADS—depression qualitative				*p* = 1.000^b^
Borderline	30% [30/100]	30% [15/50]	30% [15/50]	
Definite	9% [9/100]	10% [5/50]	8% [4/50]	
SS questionnaire completed	85% [85/100]	86% [43/50]	84% [42/50]	*p* = 1.000^b^
Sexual satisfaction	27.8 ± 5.1 [16 ÷ 40]	28.4 ± 4.8 [20 ÷ 40]	27.4 ± 5.4 [16 ÷ 37]	*p* = 1.000^a^

^a^Mann–Whitney test *p* value

^b^ *χ* ^2^ test *p* value; all *p* values were adjusted for multiple testing; the results are presented as mean ± SD [range] or proportion [number of cases/total number]

### The recalled pain perception

The mean recalled pain level experienced during the previous rigid CS was 5.7 ± 2.4 points, with full 0 ÷ 10 range of responses. In the univariate analysis, a weak positive association of the recalled pain with depression score (*r*_*s*_ = 0.33, *p* = 0.008) and weak negative correlation with sexual satisfaction (*r*_*s*_ = −0.40, *p* = 0.002) were observed. The level of the recalled pain was not related to the patients’ age, and did not associate with the anxiety level (Table 3).

**Table 3:** Correlations observed at baseline (pre-CS)

	Age		HADS—depression		HADS—anxiety		Sexual satisfaction	
	*r*_*s*_	*p* value	*r*_*s*_	*p* value	*r*_*s*_	*p* value	*r*_*s*_	*p* value
Recalled pain perception	0.05	1.000	0.33	**0.008**	0.19	0.617	−0.40	**0.002**
Sexual satisfaction	−0.39	**0.002**	−0.56	<**0.001**	*r* − 0.48	<**0.001**		
HADS—anxiety	0.19	0.611	0.51	<**0.001**				
HADS—depression	0.35	**0.003**						

All *p* values were adjusted for multiple testing; statistically significant *p* values are shown in bold

### Pain perception during the study CS – group comparison

The mean pain level experienced during the current rigid CS was 5.2 ± 2.6 points, with full 0 ÷ 10 range of responses (Table 4). The level of the currently experienced pain was similar to the recalled pain level. The pre- and post-CSs provided that responses were moderately correlated with *γ* = 0.56 (*p* < 0.001). The pain perception was slightly influenced by a result of the current CS. The positive CS, i.e., with visualization of tumour recurrence or suspicion of tumour recurrence, was described as 6.5 ± 2.3, range 3 ÷ 10, and negative as 4.6 ± 2.5, range 0 ÷ 9 (*p* = 0.039).

**Table 4:** Study group scores reported after the current CS

Score	Rigid CS group		Flexible CS group		Group comparison, *p* value^b^
	Mean ± SD [range]	Post-CS to baseline, *p* value^a^	Mean ± SD [range]	Post-CS to baseline, *p* value^a^	
Pain perception	5.2 ± 2.6 [0 ÷ 10]	1.000	1.7 ± 1.2 [0 ÷ 5]	<**0.001**	<**0.001**
HADS—anxiety	7.4 ± 3.2 [0 ÷ 15]	1.000	5.9 ± 3.1 [0 ÷ 11]	<**0.001**	0.156
HADS—depression	6.1 ± 3.4 [0 ÷ 15]	1.000	5.6 ± 3.5 [0 ÷ 13]	1.000	1.000
SS	26.4 ± 5.1 [14 ÷ 35]	< **0.001**	27.1 ± 4.7 [18 ÷ 36]	1.000	1.000

Statistically significant values (*p* < 0.05) are given in bold

^a^Wilcoxon test *p* value

^b^Mann–Whitney test *p* value; all *p* values were adjusted for multiple testing

In case of flexible CS, the currently experienced pain level was much lower than the recalled rigid CS level with the mean score of 1.7 ± 1.2, range 0 ÷ 5 (*p* < 0.001, Table 4). The pain related to flexible CS was also much lower than the current pain perception in the group R (*p* < 0.001). Despite overall lowering of the responses, the recalled and post-CS provided that responses remained weakly correlated with *γ* = 0.31 (*p* = 0.032). The result of flexible CS did not influence the pain perception level. The drop in pain perception observed during flexible CS was not related to any of the assessed scores nor patients’ age (data not shown).

Analysis of covariance showed that pain perception during CS is mostly influenced by the type of the cystoscope (*β* = 0.67, *p* < 0.001), but also by the recalled pain level reflecting the patients’ pain susceptibility (*β* = 0.46, *p* < 0.001) and slightly by the current anxiety HADS level (*β* = 0.16, *p* = 0.036). Patients’ age, depression symptoms, and the CS result were not independently influencing the pain level.

### HADS in NMIBC patients

The questionnaires’ results provided before the CS procedure were considered a baseline characteristics of the patients involved in the study. The mean anxiety score was 7.2 ± 3.0 points with 32% of the study group presenting borderline anxiety and 18% definite anxiety. The mean depression score was 5.8 ± 3.5 points, with 30% borderline and 9% definite depression observed.

The HADS score results were associated and influenced by patients’ age (Table 3). There was a moderate positive correlation between anxiety and depression (*r*_*s*_ = 0.51, *p* < 0.001) and depression but not anxiety was shown to be age related (*r*_*s*_ = 0.35, *p* = 0.003).

In multivariate analysis, depression symptoms were the only one to associate with the initial HADS anxiety level (*β* = 0.51, *p* < 0.001). Patients’ age and the recalled pain did not appear to have an impact on the anxiety score.

### HADS scores: short-term response after CS with group comparison

The type of the cystoscope used for bladder examination influenced the post-CS reported anxiety. The mean anxiety HADS score provided 7–10 days after rigid CS was 7.4 ± 3.2 points, range 0 ÷ 15 and was similar to pre-CS result. In the case of flexible CS, the mean anxiety HADS score reported 7–10 days post-CS was 5.9 ± 3.1 points, range 0 ÷ 11, and was lower when compared to pre-CS level (*p* < 0.001, Table 4).

The mean depression score was 6.1 ± 3.4 points, range 0 ÷ 15 for rigid CS and 5.6 ± 3.5 and range 0 ÷ 13 after flexible one (Table 4). The score was comparable to pre-CS assessment in both groups.

The anxiety and depression HADS results assessed pre- and post-CSs were strongly correlated in both groups (Table 5), and they were not influenced by the current CS result.

**Table 5:** Factors influencing the HADS and SS scores post-CS—single effects and analysis of covariance

Score	Single correlations				Analysis of covariance	
	Group R		Group F		*β*	*p* value
Predictors	Corr. coeff	*p* value	Corr. coeff	*p* value		
HADS—anxiety						
Type of the CS^a^	–	–	–	–	0.12	0.090
Baseline anxiety	*γ*= 0.70	<**0.001**	*γ*= 0.90	<**0.001**	0.77	<**0.001**
Baseline depression	*r*_*s*_ = 0.49	**0.001**	*r*_*s*_ = 0.54	<**0.001**	0.11	0.087
Current pain perception	*r*_*s*_ = 0.24	1.000	*r*_*s*_ = 0.32	0.444	–	0.531
Age	*r*_*s*_ = 0.28	0.735	*r*_*s*_ = 0.14	1.000	–	=0.639
HADS—depression						
Type of the CS^a^	–	–	–	–	–	0.721
Baseline anxiety	*r*_*s*_ = 0.53	<**0.001**	*r*_*s*_ = 0.64	<**0.001**	0.16	**0.004**
Baseline depression	*γ*= 0.83	<**0.001**	*γ*= 0.80	<**0.001**	0.79	<**0.001**
Current pain perception	*r*_*s*_ = 0.21	1.000	*r*_*s*_ = 0.20	1.000	–	0.652
Age	*r*_*s*_ = 0.33	0.310	*r*_*s*_ = 0.38	0.093	–	0.324
Sexual satisfaction						
Type of the CS^a^	–	–	–	–	−0.15	**0.001**
Baseline SS	*γ*= 0.84	<**0.001**	*γ*= 0.84	<**0.001**	0.90	<**0.001**
Current anxiety	*r*_*s*_ = −0.45	**0.033**	*r*_*s*_ = −0.40	0.125	–	0.356
Current depression	*r*_*s*_ = −0.56	**0.001**	*r*_*s*_ = −0.44	**0.048**	–	0.632
Recalled^b^ pain perception	*r*_*s*_ = −0.33	0.480	*r*_*s*_ = −0.49	**0.015**	–	0.643
Current pain perception	*r*_*s*_ = −0.08	1.000	*r*_*s*_ = −0.55	**0.003**		
Age	*r*_*s*_ = −0.22	1.000	*r*_*s*_ = −0.37	0.224	–	0.658

Statistically significant values (*p* < 0.05) are given in bold

^a^Value of 1 for rigid CS in multivariate analysis

^b^Recalled not current pain perception was included in the multivariate model to assess the patients’ pain sensitivity and due to very strong association of experienced pain and type of CS; all *p* values were adjusted for multiple testing

When corrected for covariance, post-CS anxiety was mostly defined by the baseline anxiety level (*β* = 0.77, *p* < 0.001), but also some negative impact of the rigid CS (*β* = 0.12, *p* = 0.090) and baseline depression (*β* = 0.11, *p* = 0.087) could be observed.

Post-CS depression was described very similarly to the first assessment (*β* = 0.79, *p* < 0.001) with slight impact of the initial anxiety (*β* = 0.16, *p* = 0.004).

The experienced pain level and patients’ age did not have a direct effect on the anxiety nor depression level post-CS.

### NMIBC patients’ sexual satisfaction scores

The mean SS score reported initially by the study participants was 27.8 ± 5.1. There was a group of patients, who did not fill the SS questionnaire. They were older (SS−: 75.1 ± 4.3 years, range 69 ÷ 83 years; SS+: 67.9 ± 7.2 years, range 48 ÷ 86; *p* < 0.001) but did not differ in terms of anxiety, depression, or pain perception from other study participants.

Both anxiety and depression negatively correlated with sexual satisfaction score (*r*_*s*_ = −0.46, *p* < 0.001 and *r*_*s*_ = −0.56, *p* < 0.001, respectively). There was a weak negative association of sexual satisfaction with patients’ age (*r*_*s*_ = −0.39, *p* = 0.002).

Multivariate analysis, including age, HADS, and PP scores, showed that baseline SS score was negatively influenced by the baseline anxiety (*β* = −0.27, *p* = 0.012), depression symptoms (*β* = −0.24, *p* = 0.043), and recalled pain perception (*β* = −0.22, *p* = 0.020).

### Sexual satisfaction after CS

Mean SS score after the current rigid CS was 26.4 ± 5.1, range 14 ÷ 35, and its level was slightly but significantly lowered compared to pre-CS value (*p* < 0.001, Table 4). Mean SS score after flexible CS was 27.1 ± 4.7, range 18 ÷ 36, and its level was not influenced by the CS procedure.

SS after the current CS was not associated with pain perception (Table 5). In addition, the change in SS was not related to pain perception nor patients’ age. As it was observed at baseline, SS was negatively related in both groups to depression and in case of rigid CS, also the negative influence of anxiety on SS was observed. The patients’ age moderate negative association with SS was observed in group F (*r*_*s*_ = −0.55, *p* = 0.003), but was not observed in group R.

Sexual satisfaction scores provided that post-CSs were strongly associated with the previously described baseline SS level (*β* = 0.90, *p* < 0.001), but also a negative impact of the rigid CS (*β* = −0.15, *p* = 0.001) could be observed in the multivariate analysis.

## Discussion

In our study, we measured pain perception and changes in anxiety, depression, and sexual satisfaction levels in a homogenous group of 100 male patients who were under cyclic surveillance after TURB because of NMIBC. Differences between rigid and flexible CS were analysed in the course of the study. Our study was blinded; patients were not aware what type of CS would be performed at the time they completed the survey. We chose to assess pain, depression, anxiety, and SS levels not more than 10 days following procedure. It has been already proven that the biggest changes can be observed in a short time after surgery and differences gradually disappear over days [25, 29].

### Pain perception

Similar to our previous study, it was shown that pain sensation remembered by patients from the previous CS is high (mean 5.7 ± 2.4 points) often reaching ten points, which is quoted as “unbearable pain” [5]. The level of the recalled pain was not related to the patients’ age and HADS anxiety level; however, it was positively associated with depression symptoms.

During the current CS, it was demonstrated that pain associated with flexible CS was three times lower (mean 1.7 ± 1.2) than reported after rigid CS (mean 5.2) independently of patients’ age. In addition, no one patient after flexible CS rated pain perception for more than five points, whereas after rigid CS, eight patients rated pain as severe (8–10 points).

The recalled and current pain perception scores are correlated, even in the case of observed pain decrease after flexible CS. It suggests that patients have a given susceptibility to pain perception, regardless of a performing physician and the cystoscope type.

A rigid CS with a positive result, i.e., visualization of tumour recurrence or suspicion of tumour recurrence, was more painful than the negative one. Positive rigid CS may be more painful due to the fact that suspicious areas are always meticulously visualized, thereby increasing the amount of cystoscope manipulation and often CS time. There was no difference in pain perception caused by the flexible CS result, which also supports the superiority of the flexible cystoscope.

In addition, mental syndromes may interfere with invasive examinations resulting in greater pain perception [30]. We observed that, although the pain perception during CS is mostly influenced by the type of the used cystoscope and patients’ pain susceptibility, it is further influenced by the anxiety score but not patients’ age.

In many patients, the anticipation of pain caused by CS can be a sufficient motive not to undertake primary diagnosis or waive the cyclic surveillance after TURB. This relationship is likely to be particularly pronounced in patients with psychological disorders [31–33]. For that reasons, patients who are particularly sensitive to pain should be referred to a hospital, where it is possible to perform flexible CS. In addition, rigid CS under general/spinal anaesthesia is an option, but it is related to higher cost and higher risk of complications and cannot be performed in an outpatient setting [34].

### Anxiety and depression

It has been proved that oncological patients have increased anxiety and depression levels compared to general population [22]. It has also been shown that a large proportion of patients with BC undergoing radical cystectomy experience psychological distress during the perioperative period [35]. Our observations confirm the hypothesis that HADS scores are also higher in NMIBC patients’ population. The reported anxiety level was related to depression symptoms with no independent influence of patients’ age.

There are no available data on anxiety and depression levels measured by HADS scale in general Polish population. In somehow similar German population, the mean scores for anxiety and depression in males are 4.4 and 4.8. Using the cutoff 8+, the percentages of elevated anxiety and depression in the total sample are 21 and 23%, respectively [36]. In the case of Polish oncological patients, it was documented that in population with gynaecological and breast cancers, the average anxiety was ranging from 7.0 to 8.2, and average depression from 6.1 to 8.4 depending on disease stage and therapeutic method [37, 38]. In Polish patients with gastrointestinal tract cancers, it was found that the average severity of anxiety was 6.7 and average severity of depression was 4.8 [39].

In Polish population of testicular cancer, average severity of anxiety was 5.71. The prevalence of anxiety disorder (8–10 pt.) was 40% and anxiety syndrome (11+ pt.) −15% in patients during chemotherapy [40].

We observed that the level of anxiety reported after a few days post-CS is positively influenced by flexible CS. This observation could be explained by the fact that less pain during CS not only allows to quickly reduce psychological tension after procedure, but also gives insights that the future surveillance can be less burdensome. What was similar with Seklehner et al. conclusions, depression levels did not change after CS notwithstanding the used method [25]. Furthermore, the anxiety and depression levels were not influenced by the current CS result. What is in accordance with earlier findings, BC diagnosis might have a limited influence on anxiety and depression short time following CS [41, 42].

Some authors state that HADS levels are “falsely” elevated before CS, as CS contributes considerable stress to the examinee. We have not observed any decrease in anxiety following rigid CS examination. However, when a less painful flexible CS was performed, the patients’ anxiety was reduced.

It is in agreement with Seklehner et al., who demonstrated that in patients who anticipated rigid CS anxiety levels were higher comparing to flexible CS [25].

### Sexual satisfaction

In our paper, we showed that sexual satisfaction is negatively influenced by the anxiety and depression symptoms. We could also observe the negative impact of pain susceptibility on SS score. Although we were not able to prove an independent influence of age on sexual satisfaction, we noted that patients who were not responding to SS questionnaire were older.

Sexual satisfaction scores provided that post-CSs were strongly associated with the previously described baseline SS level; however, a negative impact of the rigid CS on SS was observed. We observed that there was no change in SS levels after CS in patients undergoing flexible procedure. In rigid group, although there were no large differences in the general analysis, the paired evaluation of pre- and post-CS results showed a significant decrease in SS after rigid CS and demonstrated negative association with anxiety. Once more, post-CS SS was negatively related to depression. It clearly shows that rigid cystoscopy has a negative impact on patients’ sexual well-being.

It is widely known that satisfaction with sexual life is of great importance in the global assessment of quality of life made by adults [43, 44]. It helps to maintain satisfaction within a relationship and to provide considerable emotional, psychological, and physical pleasure [28]. Any malignancy of the lower abdomen and pelvis may lead to sexual dysfunction due to psychogenic or organic causes [45]. It was reported that in patients after definitive therapy for localized prostate cancer, physiologic sexual dysfunction impacted the quality of sexual intimacy, everyday interactions with women, sexual imagining and fantasy life, and self-perceptions of masculinity [46]. In BC patients’ group, reliable data regarding sexual function refer almost solely to patients treated because of muscle-invasive BC [8, 47]. Some studies show that prevalence of sexual dysfunction in NMIBC patients, which is the largest group of new BC diagnoses, is very high compared with an age- and gender-matched healthy population. What was also proven is that almost a quarter of the sexually active patients with NMIBC are afraid of harming their partner during sexual contact [6].

It was reported that rigid CS might transiently impair functional sexual performance and libido in ¾ sexually active patients [6, 29]. However, available data on CS impact on SS are meagre and do not include comparison between rigid and flexible CS.

## Limitations

Our study has some limitations. The rigid cystoscopies were not performed by the same urologist. CS, such as every surgical procedure, may be performed slightly differently among urologists. As the patient’s discomfort may (to some extent) depend on the way, the CS is performed, and it could have some effect on the results. Second, study was conducted in one university centre and on a relatively small sample. However, the group was very homogenous. There might have been some qualification bias, as we included only patients that were able to fill out surveys without the help of the third parties. We also lost some of the participants for two reasons. Twenty three patients were unable to fill the initial questionnaires, which might have resulted from strong anxiety at the admission for CS. In addition, 17 of the enrolled patients did not send the second copy of the questionnaires. It is possible that depressive patients were less willing to fill out the second copy of surveys, and, therefore, were not included in the analysis. Finally, there might have been some qualification bias, as we included only patients that were able to fill out surveys without the help of third parties. Possibly, the paper does not include a complete cross section of NMIBC patients. It is likely that the paper presents younger and also more sexually active group.

## Conclusion

Our study provides the general characteristics of anxiety, depression symptoms, and sexual satisfaction of NMIBC patients. We show the superiority of flexible CS over the rigid one in terms of pain perception, but also in sexual satisfaction or anxiety levels in male patients who were under cyclic surveillance after TURB because of NMIBC. Modern flexible video cystoscopes are characterized by excellent image quality, and wide range of active tip deflection, which allows for visualization of whole bladder mucosa, especially the sites which are difficult to access using rigid CS. The improvement in patients’ comfort together with excellent diagnostic results obtained with modern flexible cystoscopes make them a method of choice for bladder examination in male patients.
